# Exploring the factors impacting choice and quality of overnight private hospital stays and consumer perspectives on patient reported experience measures (PREMs) in Australia: a qualitative interview study

**DOI:** 10.1186/s41687-024-00755-3

**Published:** 2024-07-20

**Authors:** Krista Verlis, Kirsten McCaffery, Tessa Copp, Rachael Dodd, Rebekah Laidsaar-Powell, Brooke Nickel

**Affiliations:** 1 Sydney Health Literacy Lab, Sydney School of Public Health, Faculty of Medicine and Health, The University of Sydney, Sydney, NSW Australia; 2 The Daffodil Centre, Faculty of Medicine and Health, A Joint Venture Between The University of Sydney and Cancer Council NSW, Sydney, NSW Australia; 3 Psycho-Oncology Cooperative Research Group (PoCoG), School of Psychology, Faculty of Science, The University of Sydney, Sydney, NSW, Australia

**Keywords:** Patient reported experience measures, Decision-making, Hospital admission, Hospital report cards, Private health insurance, Qualitative

## Abstract

**Objectives:**

Patient reported experience measures (PREMs) are tools often utilised in hospitals to support quality improvements and to provide objective feedback on care experiences. Less commonly PREMs can be used to support consumers choices in their hospital care. Little is known about the experience and views of the Australian consumer regarding PREMs nor the considerations these consumers have when they need to make decisions about attending hospital. This study aimed to explore consumer awareness of PREMs, consumer attitudes towards PREMs and the utility of PREMs as a decision-making tool in accessing hospital care.

**Methods:**

Qualitative study involving semi-structured interviews conducted over the phone. Participants (*n* = 40) were recruited from across Australia and purposively sampled according to key characteristics: holding private health insurance, > 30-years of age, may have accessed private hospital care in the past year, variety of educational and cultural backgrounds, and if urban or rural residing. Interviews were audio-recorded, transcribed, and analysed thematically.

**Results:**

Four overarching themes and six subthemes were identified from the data. Major findings were that prior awareness of PREMs was limited; however, many had filled in a PREM either for themselves or for someone they cared for following a hospital stay. Most respondents preferred to listen to experience of self or family/friends or the recommendation of their physician when choosing a hospital to attend. Participants appeared to be more interested in the treating clinician than the hospital with this clinician often dictating the hospital or hospital options. If provided choice in hospital, issues of additional costs, timeliness of treatment and location were important factors.

**Conclusion:**

While PREMs were considered a possible tool to assist in hospital decision-making process, previous hospital experiences, the doctor and knowing up-front cost are an overriding consideration for consumers when choosing their hospital. Consideration to format and presentation of PREMs data is needed to facilitate understanding and allow meaningful comparisons. Future research could examine the considerations of those consumers who primarily access public healthcare facilities and how to improve the utility of PREMs.

**Supplementary Information:**

The online version contains supplementary material available at 10.1186/s41687-024-00755-3.

## Background

Variations can exist in both the quality of hospital and in the desire for choice by consumers for hospital care. Patient Reported Experience Measures (PREMs) are ‘a measure of the views and observations on different aspects of the health care services that a person has received’ [1, 2]. This includes aspects of the physical environment where the services were provided and of interactions between patient and clinician [3]. PREMs data is an increasingly useful tool for improving quality of care, aiding in healthcare delivery and can remove some of the subjectivity associated with other satisfaction measures. This is because PREMs do not look at outcomes and patients objectively report on the impact of processes on their experiences [4–6].

Patient experience scores and patient-reported indicator surveys are common tools both in Australia and globally. The development and adoption of their use by different providers and programs is growing as many national governments expand on the collection and reporting of this PREMs data [7–9]. There are also efforts underway through Organisation for Economic Cooperation and Development (OECD) to standardise and expand a Patient Indicators Surveys Programme [10, 11]. To improve value-based healthcare and to meet the healthcare needs and expectations of the public an understanding of the factors valued by healthcare consumers and information on the experiences and outcomes of care is needed [7, 11].

Australia has a mixture of public and private hospitals, and the private hospital sector has a significant role in providing specialised care with ~ 44% of Australians holding private health insurance [12]. This theoretically gives private health insurance consumers the ability to choose hospital care, which is why it is important to focus specifically on the private health system in this context. There is an in interest by private health insurers in understanding what role PREMs may have in guiding hospital choice by these consumers.

A systematic review of patient and healthcare provider perceptions on using PREMs in routine clinical care indicated some notable gaps around patient experience and perspectives of PREMs with only four studies exploring these factors [1]. Whilst studies have explored hospital staff perspectives of PREMs and their utility in hospital quality and improvement processes [13–15], there remains a dearth of literature on how publicly reported PREMs can be utilised to inform consumer hospital choice. Many hospitals have PREMs data available for prospective patients to access and some private health insurers, such as Medibank Private (Medibank.com.au) also present this information on hospitals which their members can attend. This project addresses this knowledge gap by exploring if and how consumers utilise publicly available PREMs data; if this information was or could be useful as a decision-making tool in accessing hospital care; and if or what influenced preferences or behaviours around private hospital choice.

## Methods

### Study design

This qualitative study used semi-structured telephone interviews to capture the views of Australian consumers on what factors, including an awareness and use of PREMs, guided their hospital choice. This project received ethics approval from The University of Sydney Human Research Ethics Committee (2022/686). All participants gave written informed consent prior to participating.

### Participants

Participants (*n* = 40) were recruited from across Australia. Participants were aged 30-years and older and held private health insurance. People with a recent (within past 12 months) private hospital admission were purposively oversampled. An independent research recruitment organisation (Tavener Research Group) used random landline and location-known mobile samples to contact potential participants by telephone. A series of questions were used to assess eligibility. People were excluded if they failed to meet age and insurance requirements, and overnight hospital admissions needing to be as private hospital patient. Interpreting services were not available for those not fluent in English. Interested participants were then emailed the Participant Information Statement and booked into an interview time. To gain a diverse range of perspectives, purposive sampling was utilised to ensure inclusion of participants with varying education levels, genders, geographic locations (urban and rural; states and territories), across a variety of age ranges (30–39, 40–49, 50–59, 60–69, 70+). Participants received a $50 retail gift voucher as compensation for their time. This number of participants enabled thematic data saturation to be achieved, as indicated by no further original themes being raised in the data [16, 17].

### Data collection and analysis

Interviews were conducted between December 2022 and April 2023. Interview schedules were first piloted before being finalised. Participants were informed on what PREMs were and on how individual hospitals may publicly report on patient experiences. Reporting both as a tool for consumer decision and as a role with staff internal quality control purposes (see Appendix A for full interview schedules). Semi-structured telephone interviews conducted with a qualitative researcher (KV, RL-P, BN), were audio-recorded and then transcribed verbatim with identifying information removed. Quantitative eligibility data for each participant, which included demographic and screening question data was collected by Tavener Research Group, further questions like those around health literacy and quality of life (QoL) were also gathered by the researcher at the start of the interview. This data was compiled into an Excel spreadsheet. The use of QoL measures, physical wellbeing and health literacy measures have been single items adapted from other questionnaires and surveys (e.g., QLQ-C30, SF-36) and been validated and have demonstrated high internal consistency in previous studies involving healthcare consumers [18–21]. Interviews averaged 31 min (range 19–55 min). Transcripts were thematically analysed in NVivo 12 Software [22] using a Framework Analysis that identified recurring themes and data patterns [23, 24]. The framework approach was chosen as it facilitates comparisons within and between cases and would allow for sub-group analyses (if required). It also provided a clear approach to managing large numbers of participants. Analysis was conducted according to the five stages outlined by Ritchie and colleagues [23]. Three members of the research team (KV, RL-P, BN) were involved in the development of the coding framework to ensure consistency and methodological rigour with any disagreements resolved prior to coding and analyses of all transcripts. Participant demographic data is descriptively reported.

## Results

Forty individuals (19 men and 21 women) participated in the interviews. Characteristics and QoL of participants are described in Table 1.

**Table 1:** Participant demographic and QoL characteristics

Characteristic	Overall (*n* = 40) *n* = (%)
**Age**	
30–39	11 (27.5)
40–49	10 (25.0)
50–59	7 (17.5)
60–69	7 (17.5)
70+	5 (12.5)
**Gender**	
Female	22 (53.7)
Male	19 (46.3)
Non-binary	0
**First language**	
English	34 (82.9)
Other	7 (17.1)
**Rural or Urban**	
Urban	35 (87.5)
Rural	5 (12.5)
**Highest education qualification**	
Left school before Year 12	2 (5.0)
Year 12/High School Certificate	5 (12.5)
TAFE Certificate or Advanced Diploma	13 (32.5)
Bachelor’s Degree or above	20 (50.0)
**Health literacy**	
Never	27 (65.9)
Rarely	12 (29.3)
Sometimes	2 (4.9)
Often or Always	0
**General health**	
Very good	6 (15.0)
Good	21 (52.5)
Fair	10 (24.4)
Poor	3 (7.5)
Very poor	0
**Quality of life (QoL)**	
Excellent to Very good (7 − 6)	18 (45.0)
Good (5)	16 (40.0)
Fair (4)	5 (12.5)
Poor to very poor (1-3)	1 (2.5)

Thematic analysis of interview data identified four overarching themes: (1) General attitudes and experiences towards private hospitals and considerations when choosing a hospital; (2) Researching the hospital or specialist; (3) Attitudes towards PREMs; and (4) Preferences for PREMs.

### Theme 1: General attitudes and experiences towards private hospitals and considerations when choosing a hospital

#### 1a. Presence of choice and factors considered when attending hospital

In most instances, attending a private hospital by participants in this study was due to a planned procedure. Most participants reported that they either did not have a choice or preference of which hospital. Hospital options more often were dictated by the consulting doctor. When a choice was possible, the factors influencing the choice of hospital were often cost, location, timeliness and experience. If it was an emergency, often a public hospital was initially attended and the participants either transferred to a private hospital from there (e.g., to attend rehabilitation) or attended the public hospital as a private patient. Factors that made for a positive or negative hospital experience included the experience of care, and available services and amenities. These factors are summarised in Table 2 (expanded table provided in Appendix B).

**Table 2:** Presence and absence of choice and factors considered when accessing hospital care

Factor	Supporting quotes
Presence or absence of choice	• *“I mean*,* you got told that when you’ve got private health insurance you’ve got choice. But really*,* it depends on where your surgeon operates and if they have privileges at a particular hospital.” (F*,* 40–49*,* urban)*
Technical or medical factors	• *“[The doctor is] basically saying to me there’s a chance of a better outcome at that hospital because he’s got what he needs for him to do his job best. Of course*,* that’s compelling to me. Of course*,* I’m going to go*,* “Oh well*,* if you think*,* we’ll just drive another 15 minutes*,* if you’re telling me that that’s where you can do your best work.” (F*,* 50–59*,* urban)*
Doctor related factors	• *“My feeling is if the GP is sending you somewhere*,* they’re doing that for a reason. I mean I’m sure it’s their buddy*,* but I’m sure they’re not going to send you to someone terrible… I don’t think most doctors*,* they’re in it to*,* I don’t know*,* to scam people” (F*,* 50–59*,* urban)*
Experience of self or others	• *“I was comfortable with the hospital where I was going to because I had been there before a number of years ago. But I had also had family and friends who had been at that hospital as well.” (M*,* 50–59*,* urban)*
Costs	• *“I would also look at the out-of-pocket expenses related to that particular hospital*,* and if it was a preferred hospital for my insurance provider. Obviously*,* that’s a big thing… nowadays it’s probably a bigger thing than anything else… It doesn’t really matter how much you might like to have a particular doctor*,* but if you can’t afford that doctor or that hospital*,* then you can’t go there.” (F*,* 50–59*,* urban)*
Location	• *“Proximity…The distance to my house would probably be my biggest thing just so that like I said*,* I still at least get to be close to my family*,* would be the overriding thing. But like I said*,* I don’t have that choice which is a bit disappointing” (F*,* 40–49*,* urban)*
Timeliness of treatment	• *“I’d been booked in at a few different hospitals and [I was] just basically following the surgeon to wherever was available just because I wanted to get them out as soon as I could.” (M*,* 30–39*,* urban)*
Person-centred care	• *“Sometimes the smallest thing will make the biggest outcomes for you as far as improving your experience. [These include things like when] you feel listened to*,* you feel cared for*,* you feel validated [by hospital staff].” (M*,* 40–49*,* urban)*
Access to private room	• *“Maybe privacy [a single room]. Because when you’re going private [healthcare]*,* I think there’s an expectation that you’ve got some privacy as well.” (M*,* 30–39*,* urban)*
Amenities and services	• *“That’s where I’m referring to that experience of it*,* so things like a comfortable bed and being able to have post recovery*,* the care in terms of having someone being able to attend if you need it*,* food*,* drink availability and entertainment*,* having a TV.” (M*,* 30–39*,* urban)*
Hospital size	• *“I felt it’s a larger [hospital*,* this is better]– because I’d tried other [smaller] ones and they just didn’t have enough [services].” (F*,* 30–39*,* urban)*

### Theme 2: Researching the hospital or specialist

#### 2a. Research on the doctor and hospital

More emphasis appeared to be placed on researching the specialist/surgeon than on the hospital, with half of participants researching their specialist prior to their procedure. The nature and severity of the procedure was raised as a factor in whether a person would seek out information on a doctor or not.

*"I didn’t do any research about the hospital. No*,*… I just looked around for… a surgeon*…" (M, 60–69, urban)

If information was sought about the doctor, it was generally around their expertise as shown in clinical outcomes and (negative) reviews. Others just checked to “*see [if] anything major [had] happen[ed]”*. Those participants who did not search out information on their doctor, said they trusted that the doctor would do a good job and didn’t “*want to feel like [they] can’t trust them in doing their job so… [they] don’t want to read about that*”. Another participant said they wouldn’t look anything up, as they “*just trust that the doctor is going to do the right thing by [them]*”.

Less people reported researching the hospital. Those who looked up information, said they checked out *specialities* and who the specialists were on staff, the amenities or services offered and read reviews. If it was to be a long stay, *“[they] wanted to know are the facilities what [they’d] feel [they’d] like to recover in.*” Some tried to speak to people who had previously attended that hospital. Those who had investigated the hospital beforehand indicated they would do so again in the future. Those who previously hadn’t investigated indicated they might in the future depending on the reason for hospital attendance or length of stay.

*"I researched*,* checking out their website*,* seeing what their credentials are what specialists operate from there. And seeing if it’s a world class leading facility within Australia. It firms up my decision making to use it. It has that sort of*,* yeah*, *a strong reputation in amongst other offerings*." (M, 60–69, urban)

There was some concern about the bias of people who post reviews online about a hospital with participants describing how those who have had negative experiences are more likely to post and therefore needing to take reviews “*with a grain of salt*”. There were additional concerns that those living in regional areas where there is only one choice of hospital and seeing bad reviews could raise anxiety about attending. There was a recognition that there needed to be a balance of reviews, and participants thought perhaps hospitals needed to be more active in encouraging those who had positive experiences to also lodge their experience.

*"Yeah*,* [bad reviews] that genuinely feeds your anxiety and you don’t need to feel that when you’re going into hospital*,* which is why a lot of people won’t read it.*,* I do like to read through things and just think*,* ‘Okay*,* well*,* I need to prepare myself for maybe that happening’*, or whatever. But sorting out what’s a genuine complaint and what’s someone who’s maybe had to wait five minutes longer for a nurse to get to them and then decided that everything was s***, (F, 50–59, rural)

#### 2b. Ways of researching

Google was a common tool for searches on doctors, but some expressed concerns around interpreting the reviews posted to Google and making decisions based on the experiences of others.

*“it’s really hard to form a decision just based on other peoples’ experiences.* (M, 30–39, urban)”

Some doctors had their own websites and actively encouraged consulting patients to “*look them up*”, with some having a “*policy of disclosing that information*”. Some participants described consulting specific websites dedicated to medical professionals (e.g., Rate your MD, White Coat) before their stay, or sometimes afterward, due to their experiences. However, there were issues raised with possible interference in these reviews to control the narrative of the review.

Some participants had friends or family who recommended a particular doctor for a particular procedure/treatment and described how this recommendation was sufficient to decide. Commonly, the participants’ GP recommended a particular specialist. For some though, several sources of information were consulted in their decision making.

*"…they suggested this particular clinic and one of the doctors there - surgeons there - and I trust my GP…And I did a bit of research online and he seemed to be a decent surgeon."* (F, 40–49, urban)

Information on the hospital was obtained from a variety of sources. This included what was presented on the news, through word of mouth from family or friends, via online searches, hospital websites or from Facebook forums. Some obtained information from their GP or directly from the treating specialist/surgeon. Those who lived nearby a facility said they would consider driving by and/or trying to visit a ward prior to an admission if they were unfamiliar.

### Theme 3: Attitudes towards patient reported experience measures (PREMs)

Most participants did not know what PREMs were prior to this study. Despite this, after defining PREMs, many had either completed one in the past, either for themselves, or on the behalf of a family member.

#### 3a. Perceived benefits of independently reported or publicly available PREMs

Some participants were interested in knowing the experience of other consumers. Participants described how reading these experiences could help to identify any *red flags* about a hospital and/or doctor and could help people to “*get the information they need so they can make informed decisions*” about where to attend care. Some thought PREMs could help better prepare someone to enter hospital and could put them at *ease*. PREMs were thought to be especially relevant to those who’ve “*never really had a great deal of experience choosing a hospital…[PREM reporting] can make a choice easier*” or for those coming from another area to attend hospital care and again might be unfamiliar.

*"I definitely want to know what the experience of previous patients has been. And*,* like I said*,* I hear from friends and family and I read the media*,* but if there’s some kind of official reporting mechanism that’s available to future patients. Yeah*,* I would absolutely want to know how they felt things went for them."* (F, 30–39, rural)

There was some acknowledgement that survey responses can be variable and “*subjective… but [they] think there’s some grain of truth*” in what people report, even if the content was negative. Some thought that PREMs might be a more trustworthy form of viewing reviews of different hospitals because it was independently analysed and presented. Therefore, any ulterior motives for suggesting a particular hospital could be overcome by an “*independent assessment of the hospital*”. Concern about the bias of doctors in recommending certain hospitals was raised, as some participants had learned before or “*afterward that [their doctor had] financial dealings with the hospital [they attended]*.” Those participants indicated a preference for *“something that’s out in the open and patient-based*” with the independence of PREMs public reporting seen as a benefit.

*"Because you think to yourself*,* I wonder if the specialist here*,* they’ve got some link with the specialist up there and it’s not necessarily my medical care that’s being looked after*,* but their own interests."* (F, 70+, urban)

The potentially different drivers of care in public versus private for-profit systems may also contribute to the PREMs utility by putting these patient care factors as metrics of performance.

One participant stated, *“Private hospitals are a business [and are] there to make money [and] specialists… pay to operate out of them… So a rating system would be excellent to have [to] help guide people to make a better selection… [and remove] some of the troublesome issues in the [private healthcare] industry.”* (M, 30–39, urban)

#### 3b. Concerns or drawbacks of PREMs

Participants indicated that they would want assurances that the PREMs data was independent and from patients themselves and not “*come from the staff that runs these places*”. Independently collected, collated, and presented data would increase the appearance of impartiality. It was also acknowledged that for PREMs to be useful they needed to provide a “*standardised unit of measure that every hospital has to be measured against. [So that] you can compare apples with apples*”. Understanding of the response rate was also flagged by a few participants to determine how much weight to give to the review.

An overall review of a hospital was often viewed as not being as meaningful due to nuanced variations within hospital departments and by admission type, which could be quite significant and relevant. For example, a person admitted to hospital for mental health reasons may not require pain medications, and their support needs may differ. To be meaningful the metric would need to reflect these differences.

*"…There can be this overall view of the hospital*,* and the way that the hospital is being managed and run*,* but then to know what’s happening in a particular department… because some departments of a hospital might be much [better] quality than others. So [for example] just the fact that Hospital X has a four-star rating*,* doesn’t mean that that the neurology ward is going to be four stars."* (F, 70+, urban)

Some thought PREMS were “*generally a good thing*,* but you need to consider that [the grade/review] could also be influenced by other factors*” such as pain and discomfort. There were concerns about the subjective nature of experience and that people more likely to complete PREM surveys are those who have complaints, and those complaints might be based on experiences outside the control of the hospital (e.g., staff can only do so much to make someone comfortable post-surgery). Some believed reporting of experiences may not be reflective or meaningful because of these issues.

"[*I’m] inclined to take [these types of reported measures] with a grain of salt*,* just because [I] feel like there’s a natural bias in how if you respond to those feedback things in a certain [negative] way."* (M, 30–39, urban)

Not all participants were sure how helpful or practical PREMs would be for patients and would only consider them as “*another tool*” but wouldn’t “*put a lot of weight [on them and they] wouldn’t guide [their] decision…*”. Some participants said PREMs might only be something they consulted if they were attending hospital for a longer stay or they “*if there was any part that [they] were umming and ahhhing [about].*” In general, many participants would ultimately trust the direction of their specialist/surgeon to attend a particular hospital.

*"No*,* it wouldn’t help me make a decision*,* because at the end of the day I would still consult the doctor*,* and based on the doctor’s recommendation I would prefer [to attend where they suggest]."* (M, 30–39, urban)

Having the ability to add comments along with the metrics was suggested as a means to overcome some of the concerns around negatively biased reviews. Some thought that giving a metric is not particularly meaningful without *context* and *justification*. One participant noted the need to have “*the ability to actually read some type of text rather than just having a 0–10 score*” as a number is not really meaningful in isolation, what was the “*underlying reason*” for that score.

*"So*,* for me*,* numbers don’t really mean that much. You do see the numbers and I think*,* ‘Well*,* how did they come to that number? What was it? And what part of it made them give them that rating? Was it the service*,* was it the room*,* was it the location?’ And it needs to be done in category."* (F, 50–59, urban)

Some thought the feedback was more important to hospitals in understanding patient experience as to identify issues and recognise when things are done well, rather than by consumers to select a hospital.

*“[PREMs are] more important to the actual care providers themselves*,* [as] they’re the things that they should be looking at and thinking about and acting upon*” [and could be helpful in showing] “*what the health care providers need to do*,* but equally [could be used to give] recognition…to the staff who are working really hard.”* (M, 30–39, urban)

Participants were also unsure anything was done by the hospitals with the collected information even when the hospital had stated they would follow up.

### Theme 4: Preferences for patient reported experience measures (PREMS)

#### 4a. Content of what is important prior to a hospital admission

Those seeking information wanted as much information up-front about their hospital stay as possible, including available services, and specialists on staff. A *general overview* of the hospital with an *about section* to understand the *philosophy* and *history* of the organisation could allow someone to get “*a bit of a vibe*” of the place beforehand and presented a “*good opportunity*” for hospitals to promote themselves in a way that would make people “*feel a lot less anxious and freaked out*” about attending. There was an interest for more general information on what private health funds are accepted, any out-of-pocket or excess costs and what services and technology were available. Information on the admission and discharge procedures and what assistance is available during these processes was suggested. This could facilitate ready comparison if desired, allow for better preparation and could help customers ask better questions before their stay. There was also an interest in pictures of the general appearance of the facility and of patient rooms and the availability of services like psychological support and rehabilitation services.

*"To have a number of costs*,* quotes provided for you upfront on different hospitals. So at least you can look at the cost of each as well…"* (M, 50–59, rural)

Many desired reassurance that they would have access to a private room during their stay. Other issues where information was desired included parking availability for visitors with associated cost, food quality and type of available catering. Additionally, noise levels on wards, the cleanliness of the facility and how often rooms and facilities were cleaned, room presentation, and even the availability of heating in patient rooms. The newness and the feel of the hospital and comfort of the hospital stay were also identified.

Patient care was further flagged by many participants. This related to the attentiveness of nurses and doctors, the friendless of the staff and feeling listened too and cared for, as well as information on nurse-to-patient ratios.

Participants also sought information about the occurrence of more serious clinical issues such as unexpected deaths, hospital-acquired infections, incidences of malpractice and patient neglect, and adverse events. The statistics on types of surgeries performed and the success and error rates were also mentioned when reading about a hospital. For example, if a hospital performed a certain surgery quite frequently (and successfully) it was thought that this would give more confidence in attending that hospital for that condition.

*"…unexpected deaths*,* infections*,* lackadaisical staff*,* bad food*,* things like that… A list of hospitals which do offer that service*,* and then I would have to do my own– and if the doctor operated from those hospitals."* (M, 60–69, urban)

#### 4b. Functionality of presented PREMS data

When asked about their preferences for how PREMs could be best displayed for health consumers selecting a hospital. Participants consistently preferred an online, readily accessible platform as the preferred format. Ideally this would have a search function by state and then by hospital name. Some thought an email containing information on the hospital or a link to this information might be useful for those not comfortable navigating online. Commonly, participants indicated that they would like to be able to compare between hospitals, with a comparison of two or three seen as ideal as more might overwhelm. Not all wanted to be able to compare though, preferring to look at hospitals in isolation. Further suggestions on factors related to the functionality and presentation of PREMs data is provided in Table 3.

**Table 3:** Factors related to the functionality and presentation of the PREMs data

Factor	Supporting quotes
**Department and admission type**: Department level or by admission type review, not just hospital wide review.	*“I think… you can give a rating but it might not be particularly relevant to that situation… [for example] a mental health admission*,* I don’t necessarily want somebody to come and manage my pain or anything like that. Where I might be thinking that it’s not necessarily relevant*,* so*,* I’ll go*,* no*,* not often*,* well*,* you don’t need often”. (F*,* 40–49*,* urban)*
**Understandable**: Use of plain English and available other languages	*“As long as the information was able to be interpreted by a layperson… [So the use of simple English*,* straightforward]*,* without all the medical jargon.” (F*,* 30–39*,* rural)*
**Accessible & comprehensive**: Limited text with ability to obtain more information if desired	*“I like to read information*,* but I am quite visual. So*,* I’d love to have visual charts*,* that tells you the percentage of positive outcomes. But then*,* the deep dive into the information are linked to another page. So*,* you’re not overloaded with information if it’s not relevant*,* or you don’t care too much about a certain section of it.” (F*,* 40–49*,* urban)*
**Design**: Use of infographics and graphs with informative headings/titles	“*I am pretty visual*,* and I tend to skim-read things. I guess it would be great if there were like graphs and I don’t know*,* if the information can be aggregated so they say like 20% of people gave this score for this question or something like that.” (F*,* 40–49*,* urban)*
**Pro comments**: Reviews / comments to accompany grading to provide context	*“Because I do like reading personal experiences and why they’re rating something that way. Because sometimes people rate stuff and it’s just absolutely ridiculous. I like to be able to see the reasoning behind their rating. Because they could be rating something like a one*,* but then their reasoning behind*,* it’s just not justified.” (F*,* 30–39*,* urban)*
**No comments**: Some did not think providing comment was a good idea	*“The scoring that I’ve seen recently is okay*,* but I don’t know that putting a review under it is probably a good idea. I think a scoring thing is fine*,* but I don’t know that writing reviews is such a good thing.” (F*,* 30–39*,* urban)*
**Recent**: Updated regularly	*“[PREMs website should be] constantly updated with information rather than stale or old information” (M*,* 50–59*,* urban)*
**Independent**: Independently collected and presented to avoid any biased reporting	“*I think if it’s on a hospital website*,* people don’t have confidence that it’s going to be accurate because they’re just going to say good things about themselves.*” *(M*,* 40–49*,* rural)*
**Freely available**: Some disagreed and thought these metrics should be available	“*Transparent…publish this data freely on [the hospital] website” (M*,* 40–49*,* rural)*
**Sample number**: Number of people represented in any scores	*“How many people does it represent… would be a determining factor as to how much weight I gave it… Because yeah*,* if you go buy something from a shop… and you see that it’s got a one-star rating*,* if only one person has rated it*,* then it means something different to if a 100 people have given it a one-star rating.” (F*,* 40–49*,* urban)*
**Benchmark**: Known benchmark or standard used for comparisons	“…*where you’re actually comparing the information between like hospitals” (M*,* 60–69*,* rural)*
**Hardcopy**: Available in hardcopy format and/or in format that can readily printed	“*Some people are wanting*,* you know*,* a hardcopy…[so that they can] maybe then give it to a family member and say*,* ‘What do you think about this?” (F*,* 50–59*,* urban)*

## Discussion

This qualitative interview study of Australian adults who hold private health insurance provided diverse and sometimes contradictory views and opinions on accessing hospital care, reflecting a range of experiences and preferences. Consistently, private insurance allowed for choice of doctor, with this doctor often providing different hospital options. Previous hospital experiences resulted in strong participant attitudes towards selecting a hospital, with the experiences of friends, family and doctor recommendations being of high importance. Cost and wait-times would override location for many participants, though location near home was often important. However, access to the doctor of choice sometimes resulted in all these factors being secondary to treatment by that preferred doctor.

Prior to participating in this study, only one participant had consulted PREMs data outputs prior to a hospital stay. There was a lack of awareness among participants about the availability of PREMs data, and PREMs data was not considered in hospital decision-making by these consumers. When asked about how they may use PREMs in future hospital decision-making, many believed it should be a supplementary tool. There was consensus among participants that PREMs should be unbiased and accessible.

A previous Australian study that examined private hospital choice and public performance reporting [25] revealed similar findings to this study. Like PREMs, most participants were unaware of public performance reporting, and their choice was influenced by the doctor [25]. Similarly, costs were also a major consideration and something that participants in both studies wanted to be publicly available [25]. Price is also a major factor in hospital choice in the United States (US), though it has been shown that patients in the US were more likely to choose lower-quality but higher-cost hospitals if these hospitals were owned by their physician [26]. This was found, at least with regard to quality with a patient in this study, who chose to attend their surgeon-owned hospital but then experienced what they felt were poorer-quality services (e.g., lack of heating).

Another Australian study also found that trust was associated with reputation of the doctor and the hospital and that reputation of both were key drivers of trust with a good reputation equating with higher quality of care [27]. This was clearly noted in several participants in this study also, whereby these participants wanted to attend hospitals and have treatment from doctors with good reputations that they could trust. Accessing online platforms that provided reviews was something that some participants in this study undertook and has been shown to be common in the US with social media also influencing hospital choice [28]. Concerns around issues like the negative bias of ratings, the inability of doctors/hospitals to respond to negative reviews, and the anxiety that these ratings could cause to those with minimal options found in this study have been raised in other studies also [29, 30]. To overcome some of these concerns, rating websites in the United Kingdom (UK) for example, have comments sections available but these sections are moderated and the opportunity for response by physicians is available [31]. The desire for comments to interpret PREMs ratings were something that some participants in this study also thought helpful.

Further factors considered by Australian consumers when choosing hospital care were similar to many of those reported in studies elsewhere in the world. For example, for many, hospitals nearer to home were preferred, but people would compromise on location to access more timely care [32–34]. Additionally, considerations of facility standards, cleanliness, friendliness of staff, car parking availability and food quality and amenities have been raised in the literature [34, 35].

This study also found that previous hospital experience was a strong influencer on future hospital choice, and this was also shown in other studies that indicated past hospital experience played a significant role in consumer hospital choice [32, 34, 35]. Those studies by King et al. [34] and Dixon et al. [35] also found that seeking advice from friends and family can influence hospital choice, but an Italian study found this advice may be misleading and might ultimately lead to consumers ending up in lower quality hospitals [32]. That study indicated that lack of formalised websites or information like hospital ratings contributed to this poorer quality information and to a lower degree of competition between hospitals [32]. These findings give strength to formalised or independent reporting of PREMs metrics in a readily understood manner so that quality information can be accessed and used to compare hospitals by consumers. Providing consumers choice and the ability to decipher clinical quality differences between hospitals has been shown to decrease patient mortality and increase patient welfare [36]. However, these quality metrics may be challenging for consumers to understand though [37].

Ultimately this study’s findings reflect what is seen by studies from other countries. That a great deal of variation exists in what consumers may consider when choosing hospital care. The degree of choice or ability to respond to differences in quality information possibly was limited by items such as health insurance, doctor referrals and emergency versus planned procedure [37, 38]. When creating websites to report on different experience metrics, ideally an understanding of what factors that are considered important by consumers should be determined so that they can be properly represented and reported [34].

### Strengths and limitations

To the best of our knowledge, this was the first in-depth qualitative study in Australia to report on consumer views on PREMs and preferences for private hospitals. We recruited a diverse range of adults varying in educational backgrounds, age ranges and across geographic location. As this study focused only on those people who hold private health cover, this automatically created selection for people who had the financial means to pay for this cover and a belief in the private healthcare system, presenting a limitation on this study. It is recognised that further awareness amongst the sample may have provided additional views on PREMs; though this study did aim to explore if and how PREMs data is used. Additionally, despite our efforts most participants were in or around major urban centres, and participants who were unable to converse in English were excluded meaning our findings may not reflect the views of non-English speakers. Health literacy was high amongst participants; however, this study tried to account for this by including people with a range of educational backgrounds.

### Recommendations for PREMs

To improve PREMs utility, participants consistently suggested department level versus hospital level reporting of results due to the variations found between departments and admission types within a single hospital. This would allow for a better appreciation of the nuanced differences. Additionally, many thought written feedback would provide the context behind PREM grading, allowing the reader to then make up their own mind about the overall grading given. Providing transparency in the methods used and the number of participants who are included in summary data was suggested to provide further confidence in the data. Simple English and having information available in other languages, the use of infographics and ability to look for more information was viewed as important to capture for those with different information preferences and to address equity issues in culturally and linguistically diverse people and in those with varying health literacy [39, 40]. To overcome barriers in understanding of metrics such as statistics on different measures, the use of graphs and percentages are appropriate [34]. The role of hospital report cards in decision-making of Australian consumers and promotion of publicly available PREMs should be considered.

Costs as they relate to a hospital stay, any procedures, for doctors and other health care professionals involved in care, were major considerations. With the current cost of living crisis facing many Australians, cost will undoubtedly become more important [41]. Transparent and up-front costs would allow for ready comparisons, permit budgeting, and facilitate informed decision-making if a person has a choice between hospitals. Obtaining doctor costs (e.g., consultant and anaesthetist) ahead of time was consistently requested by participants as these represented a major out-of-pocket expense even with private insurance.

### Conclusions

Findings from this study indicate that while PREMs were considered a possible tool to help assist in the decision-making process. Previous hospital experiences, the doctor and knowing up-front cost are an overriding consideration for consumers when choosing their hospital. Consideration should be given to format and presentation of PREMs data to allow for easy understanding and meaningful comparisons. Future research could consider the views of those who do not hold private insurance and who primarily access public healthcare facilities as well as improving the utility of PREMs.

### Electronic supplementary material

Below is the link to the electronic supplementary material.

Supplementary Material 1

## Data Availability

The datasets used and/or analysed during the current study are available from the corresponding author on reasonable request.
