# Supplementary material for: Exploring the factors impacting choice and quality of overnight private hospital stays and consumer perspectives on patient reported experience measures (PREMs) in Australia: a qualitative interview study
Source: J Patient Rep Outcomes. 2024 Jul 20;8:75. doi: 10.1186/s41687-024-00755-3 (PMC11264639; doi:10.1186/s41687-024-00755-3)
Supplement: Supplementary file 1 — Supplementary Material 1 [file 41687_2024_755_MOESM1_ESM.docx]

**Appendix A**

**Consumer Interview Schedule**

**Group A: Non-Medibank member with NO recent hospital admission**

**Introduction**

Thank you very much for taking the time to do this interview. The main purpose of this interview is to better understand what factors you consider when accessing hospital care. Specifically, we would like to understand if patient reported experience measures (PREMs) are something you are familiar with and how these might be best used by you and communicated to you.

The interview is fairly informal in terms of structure, so if you have any questions or additional comments to add throughout the interview, please feel free to do so. It should take 30-45 minutes.

I have received your consent form. Thank you for completing that and sending it through.

Also, as mentioned in the Participant Information Sheet this interview will be audio-recorded. This is just to ensure that we have an accurate record of your responses, and everything you say will be kept strictly confidential.

Do you have any questions before we begin?

Okay, so I will start the audio-recording now.

**Consumer Characteristics**

Now just before we start talking about your experience and views, I’ve got a few quick questions to help me know a little bit more about you generally, other than what you have previously provided.

1. Could you please confirm your postcode?
2. How often do you need to have someone help you when you read instructions, pamphlets, or other written material from your doctor or pharmacy?
3. Never
4. Rarely
5. Sometimes
6. Often
7. Always.
8. In general, would you say that your health is:
9. Very poor
10. Poor
11. Fair
12. Good
13. Very good

1. How would you rate your overall quality of life during the past week?

1 (very poor)     to         7 (excellent)

**Hospital Experience**

1. In the past, have you ever had to attend hospital in Australia?

**If no**, move to Awareness of PREMs section.

1. *When were you in hospital?*
2. *Why were you in hospital?*
3. *Was it a private or public hospital?*
4. *How long did you spend in hospital?*
5. *How did you feel about your experience at the time?*
6. *Has that changed at all? How do you now feel about your experience?*

**Hospital Selection**

1. Did you have a choice in the hospital you attended?
2. In general, how much choice do you feel that you have in hospital selection?
3. What factors influenced the choice of hospital where you will receive your treatment?

*Prompt: Medical staff? Family / Friends experiences? Online information?*

1. What information is/would be important to you when choosing a hospital to attend?

*Prompt:* *Out of pocket costs, clinical information like their doctor, Patient Reported Outcome Measures (PROMS*) etc?*

1. *It is likely that many people will not have had a choice in hospital, their doctor will have told them the hospital to attend or given them a choice of two options.*

Would you like to have more choice in hospitals?

1. When would you like to get that information?
2. What type of information would be helpful to you in making your choice?
3. Do you/would you prefer to attend a private or public hospital? Why is that?

*Prompt: Quality of care? Timeliness of treatment?*

1. What do you think has influenced this preference?

**Awareness of PREMs**

**
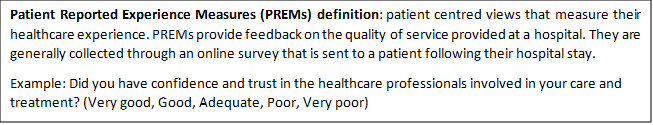
**

12. Do you know what Patient Reported Experience Measures (PREMs) are? [provide definition]

1. **[If they were unaware of PREMS]** Do you think you would use this type of information in choosing a hospital in the future? Do PREMs sound helpful to you? Why or why not?

**Use of PREMs**

1. **[If have been in Australian Hospital]**

When you were previously admitted to hospital, did you seek out any other hospital or patient reported measures or information prior to your admission? (e.g., Australian Hospital Patient Experience Question Set (AHPEQS)? Or Australian Bureau of Statistics Patient Experience Survey (PES)?)

*Was there any other information you accessed to help you decide what hospital you went to?*

1. How do you think you would most like to receive or view PREMs information in the future? 
   **Or** if you haven’t used PREMs information, and you think you would like to use it in the future, what is your preference for viewing or receiving PREMs information?
2. Online website
3. Email
4. Mobile text message
5. Paper flyer in the post
6. Other?
7. How do you think PREMs information should be presented? What would make it easy to understand? 
   *Prompt: Are you a visual person? Do you like to read information?*
8. Graphics and text mix
9. Graphics only
10. Text only
11. Verbal
12. Other

**Closing**

**Consumer Interview Schedule**

**Group B: Non-Medibank member WITH recent hospital admission**

**Introduction**

Thank you very much for taking the time to do this interview. The main purpose of this interview is to better understand what factors you consider when accessing hospital care. Specifically, we would like to understand if patient reported experience measures (PREMs) are something you are familiar with and how these might be best used by you and communicated to you.

The interview is fairly informal in terms of structure, so if you have any questions or additional comments to add throughout the interview, please feel free to do so. It should take 30-45 minutes.

I have received your consent form. Thank you for completing that and sending it through.

Also, as mentioned in the Participant Information Sheet this interview will be audio-recorded. This is just to ensure that we have an accurate record of your responses, and everything you say will be kept strictly confidential.

Do you have any questions before we begin?

Okay, so I will start the audio-recording now.

**Consumer Characteristics**

Now just before we start talking about your experience and views, I’ve got a few quick questions to help me know a little bit more about you generally, other than what you have previously provided.

1. Could you please confirm your postcode?
2. How often do you need to have someone help you when you read instructions, pamphlets, or other written material from your doctor or pharmacy?
3. Never
4. Rarely
5. Sometimes
6. Often
7. Always.
8. In general, would you say that your health is:
9. Very poor
10. Poor
11. Fair
12. Good
13. Very good
14. How would you rate your overall quality of life during the past week?

1 (very poor)     to         7 (excellent)

**Hospital Experience**

1. Can you tell me about your most recent private hospital experience? 
    *Prompts if needed:*

*When were you in hospital?*

*Why were you in hospital?*

*How long did you spend in hospital?*

*How did you feel about your experience at the time?*

*Has that changed at all? How do you now feel about your experience?*

Have you had to stay overnight in hospital before your most recent experience?

1. *Yes – was it at this same hospital? Or a different hospital? How long ago? How did it compare?*
2. *No – move to Hospital Selection section*

**Hospital Selection**

1. Was the private hospital you attended recently in your local area? Or did you have to travel to access care?
2. What was the name of the hospital you attended?
3. Did you have a choice in the hospital you attended?
4. In general, how much choice do you feel that you have in hospital selection?
5. What factors influenced the choice of hospital where you received your treatment?

*Prompt: Medical staff? Family / Friends experiences? Online information?*

1. What information is/would be important to you when choosing a hospital to attend?

*Prompt:* *Out of pocket costs, clinical information like their doctor, Patient Reported Outcome Measures (PROMS*) etc?*

1. *It is likely that many people will not have had a choice in hospital, their doctor will have told them the hospital to attend or given them a choice of two options.*

Would you like to have more choice in hospitals?

1. When would you like to get that information?
2. What type of information would be helpful to you in making your choice?
3. Do you/would you prefer to attend a private or public hospital? Why is that?

*Prompt: Quality of care? Timeliness of treatment?*

1. What do you think has influenced this preference?

**Awareness of PREMs**

1.
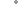
Do you know what Patient Reported Experience Measures (PREMs) are?
2. **[If they were unaware of PREMS]** Do you think you would use this type of information in choosing a hospital in the future? Do PREMs sound helpful to you? Why or why not?

**Use of PREMs**

1. Did you seek out any other hospital or patient reported measures or information prior to your admission? (e.g., Australian Hospital Patient Experience Question Set (AHPEQS)? Or Australian Bureau of Statistics Patient Experience Survey (PES)?)

*Was there any other information you accessed to help you decide what hospital you went to?*

1. How do you think you would most like to receive or view PREMs information in the future? 
   **Or** if you haven’t used PREMs information, and you think you would like to use it in the future, what is your preference for viewing or receiving PREMs information?
2. Online website
3. Email
4. Mobile text message
5. Paper flyer in the post
6. Other?
7. How do you think PREMs information should be presented? What would make it easy to understand? 
   *Prompt: Are you a visual person? Do you like to read information?*
8. Graphics and text mix
9. Graphics only
10. Text only
11. Verbal
12. Other

**Closing**

**Appendix B**

**Table 2 expanded: Presence and absence of choice and factors considered when accessing hospital care**

| **Factor** | **Explanation** | **Supporting Quotes** |
| --- | --- | --- |
| Presence or absence of choice | - Variable views on amount of choice and what constituted choice. - More than half not choosing the hospital but the doctor who dictated the hospital they attended. - Strong reliance on their doctor to guide them and *trusting* the doctor would only operate out of *good* hospitals. - Overall, preference for choice, but often out of patient control and caveats existed e.g. previous experience, location (regional/small urban centres), complex care needs. - Choice in doctor was generally more important than choice in hospital? | - *“I mean, you got told that when you’ve got private health insurance you’ve got choice. But really, it depends on where your surgeon operates and if they have privileges at a particular hospital.” (F, 40-49, urban)* - *“[Hospital A] I think would have been closer for me and easier to get to. I asked him when I met up with him in his clinic before I was ever doing any operations with him, I asked him, “Can I do it there because it’s a lot more convenient for me?” He said, “No, I can only do it in the [Hospital B] because my whole team that know me and really understand me is there. So, I can’t really do anything for you, if you want to do it there.” He wasn’t able to help me. I said, “Okay then [and attended Hospital B].” (M, 40-49, urban)* - *“I have rung specialists and gone to them, just wanting to know if I was to select this doctor as my doctor, which private hospitals does he work from.” (M, 30-39, urban)* - *“Because for me, it was based on the surgeon, you see. So it's not sort of - I wasn't shopping for hospitals per se, I was shopping for the surgeon.” (F, 40-49, urban)* - *“My surgeon operates out of three different [hospitals]. So, I get to choose which one suits me best and then we go from there.” (F, 40-49, urban).* - *“Probably not a lot of choice to be honest, especially living regionally you don't - there's not as many places you can go.” (F, 40-49, urban)* |
| Technical or medical factors | - Importance of the expertise of a particular doctor (who then dictated hospital). - Many prefer to attend hospital where the doctor felt most *comfortable* to operate / provide treatment. - Choice of doctor was emphasised depending on nature of the procedure or reason for attending hospital (e.g. childbirth or surgery). | - *“We highly recommend this person because he’s the one that we usually use and we know that he’s good with complex things like yours.” So, that’s why I ended up going with him.” (M, 40-49, urban)* - *“In terms of giving birth it's different because you need to choose from the beginning because not every obstetrician will do their practice in every hospital. It's the same again as with the other issue because they only do practice in certain hospital only.” (F, 30-39, urban)* - *“[The doctor is] basically saying to me there’s a chance of a better outcome at that hospital because he’s got what he needs for him to do his job best. Of course, that’s compelling to me. Of course, I’m going to go, “Oh well, if you think, we’ll just drive another 15 minutes, if you’re telling me that that’s where you can do your best work.” (F, 50-59, urban)* |
| Doctor related factors | - *Trust* that the doctor to only operate out of good hospitals. - GP recommendations were important for guiding doctor selection. | - *“And I just feel peace of mind that the specialist said to me, “These are the three hospitals that I attend.” And I’m thinking well, she wouldn’t be attending anything that’s not up to standard. I felt more comfortable with her telling me that “This is where I perform my surgery, so you make a choice. All three are on the same level for me.” She’s not saying, “Oh, look [name], pick that one because” - No, not at all, she was open to, “These are the three, pick whatever one you want, they’re all pretty much the same.” (F, 60-69, urban)* - *“My feeling is if the GP is sending you somewhere, they’re doing that for a reason. I mean I’m sure it’s their buddy, but I’m sure they’re not going to send you to someone terrible… I don’t think most doctors, they’re in it to, I don’t know, to scam people” (F, 50-59, urban)* |
| Experience of self or others | - Personal or family/friends experience was a very powerful influence. - News stories, community rumour, Google reviews and social media groups also influenced choice for some. - The *reputation* of the hospital and teaching hospitals were viewed favourably even without personal experience or knowledge. | - *“I was comfortable with the hospital where I was going to because I had been there before a number of years ago. But I had also had family and friends who had been at that hospital as well.” (M, 50-59, urban)* - *“there’s a hospital over the border in [state], but I wouldn’t go there. There is funny things you hear around town… But then, for the other hospital in [location], I wouldn’t go there for an emergency, just based on what’s happened in the past to people I know.” (M, 40-49, rural)* - *“And plus, that’s a known hospital – known brand in Melbourne. So that was easier. And when it’s a known brand, it’s kind of trustworthy.” (M, 30-39, urban)* |
| Costs | - Costs were a *major* or *overriding factor* consideration e.g. out-of-pocket expenses, excess, co-payments, and costs for hospitals. outside the insurers network with having to pay an acknowledged reality of accessing private hospital care. - Knowing costs and insurance coverage up-front, including discrepancies, would help in making informed comparisons and decision making. - Although, some were unconcerned about costs and happy to be out of pocket to more comfortable and access preferred doctor for others costs were a constraint and influenced what hospital they would choose to attend. - The costs to actually access the doctor (specialist/surgeon) were often more significant to the participants than hospital costs but determining these was often *confusing* and *challenging.* | - *“I would also look at the out-of-pocket expenses related to that particular hospital, and if it was a preferred hospital for my insurance provider. Obviously that’s a big thing. In fact nowadays it’s probably a bigger thing than anything else, actually. It doesn’t really matter how much you might like to have a particular doctor, but if you can’t afford that doctor or that hospital, then you can’t go there.” (F, 50-59, urban)* - *“I guess I have the privilege of being in a position where I'm quite happy to pay for quality care. That’s the most important thing to me. So, yes, I would consider out-of-pocket expenses. More important to me would be that I feel like I'm getting good quality care. (F, 30-39, rural)* - *“I automatically assumed as well, being at [Hospital C] it was going to be a little more expensive. And it was expensive, but I felt look, I either shop around – and the difficulty with it was that – and he told me as well. He told me, look, he doesn’t operate in public. My options are – because I asked him, “Look, what is my option? This is too expensive.” He said, “You go to another specialist, you find someone that operates in public and go on a very long waiting list. You go to another specialists that operates in different hospitals.”* *And I ended up choosing to just say with him and have to do that one hospital. There was no other option given to me.” (M, 30-39, urban)* |
| Location | - Location viewed by many as important if given a choice with a preference closer to home, especially for those with young children or those who did not have family/friends who could take them to/from the hospital. - Happy to travel to a less convenient location to access a particular doctor (and the associated hospital), a familiar hospital or newer facilities. - Emergency situations and transporting self, still choose to attend the closest hospital, even if a familiar/preferred hospital only slightly farther away. | - *“Proximity. Well, because it’s a regular thing, the fact that there’s a closer option to home would be better because it means that my family who - I’ve got a young daughter, and trying to get to see her, is a bit hard, trying to work it around her school and things like that, that if it was a closer travelling distance, I’d probably see her more. The distance to my house would probably be my biggest thing just so that like I said, I still at least get to be close to my family, would be the overriding thing. But like I said, I don’t have that choice which is a bit disappointing” (F, 40-49, urban)* - *“We don’t go to [Hospital D] because we’re not familiar with the area, we’re not sure about how parking works. Whereas with the [Hospital E] we’re 100% familiar with the whole area itself, so we can easily navigate where to go, we know where to park, all that. But if we went to Westmead, even though it’s a bit closer, we get completely lost. So those – that’s quite an important factor.” (M, 50-59, urban)* - *“Because I was feeling so sick, and I actually drove myself to the, I just thought I'd go to the nearest hospital and that. And I did have a choice, I did have a choice of other hospitals, but they would have been further to drive, and I wasn't [well enough to drive any further]… And, so if that ever happened to me again, I think I would just get an Uber and go to a different hospital. But my choice at the time was the closest hospital because I was feeling so unwell.” (M, 60-69, urban)* |
| Timeliness of treatment | - Timeliness of treatment was important for man, with some stating that receiving timely treatment was the *biggest factor* and the hospital itself. | - *“I’d been booked in at a few different hospitals and this just basically following the surgeon to wherever was available just because I wanted to get them out as soon as I could.” (M, 30-39, urban)* |
| Person-centred care | - Patient-centred factors were major determinants in what made an un/pleasant hospital care experience and were considered by more than half of participants when choosing to return to previously attended hospital or not in the future. - Good care was often described as being *attentive* and timely from *caring* and *empathetic* staff while uncaring staff were described as *inattentive*, *dismissive* and *unsympathetic.* - Communication was an important component and the way in which patients were addressed and spoken to and with by staff and between staff was also flagged. | - *“Sometimes the smallest thing will make the biggest outcomes for you as far as improving your experience. [These include things like when] you feel listened to, you feel cared for, you feel validated [by hospital care staff].” (M, 40-49, urban)* - *“The availability of staff, so you're not waiting for hours for something, particularly if someone has to shower you or something like that. The irregularity of staff attending to you. The way in which when, say nurses are doing their rounds and they're handing over from one shift to the next, they talk about you and introduce you. That can be important as to whether you feel like a person or if you're just an object that they're handing on.” (F, 70+, urban)* - *“But I felt with the private hospital, I felt like I was just the factory, I was just there as a factory person [as part of a conveyor belt]… when I went into operation, when I say it feels like a factory, so [when I was waiting to go for surgery] what they do is they line you up into the waiting operating theatre, then they just line the beds one after another.” (M, 40-49, urban)* |
| Access to private room | - Privacy was viewed as *super important* for many and there was often an *expectation* that attending private hospital means a private room, though acknowledge that this was not always guaranteed. - Desire for a private room often stemmed from previous negative experiences on hospital wards and being kept awake by other patients, and as *peace of mind* and feeling of *comfort).* - Length of stay was important consideration, with privacy more important for hospital stays longer than overnight. | - *“Maybe privacy. Because when you’re going private, I think there’s an expectation that you’ve got some privacy as well.” (M, 30-39, urban)* - *“I mean, that's what really sort of did upset me about that [hospital ward] experience that I couldn't really rest during the night [due to disruption from other patients], and I found it very, very frustrating.” (M, 60-69, urban)* - *“But having a single room is important if you’re going to be staying a while. If it’s just overnight then it doesn’t really matter.” (M, 70+, urban)* |
| Amenities and Services | - Just over half mentioned amenities as what made a hospital stay more pleasant and/or would be considered if given a choice about a hospital in the future. - Participants discussed newer facilities, good food, cleanliness, feeling comfortable, parking (and its costs), rehab facilities and after care, access to support and hospital affiliations with the nature of the stay and length of hospital stay influenced the desire for certain amenities or services. - Access to patient transport services to and from the hospital were a concern with these services being useful for some participants. | - *“That’s where I’m referring to that experience of it, so things like a comfortable bed and being able to have post recovery, the care in terms of having someone being able to attend if you need it, food, drink availability and entertainment, having a TV.” (M, 30-39, urban)* - *“But some hospitals are just so old and just creepy. And you just think, “It’s just not very nice.” There just seems to be a bit of difference with even the comfort of the bed. I don’t know, I just think a nicer, more calming, pleasant environment is just more uplifting for your recovery than being in a building that looks like it came out of the 1960s or something – One Flew Over the Cuckoo’s Nest type of – [thing].” (F, 50-59, urban)* - *“Transport home is another issue, like the last couple of hospitals there was no transport home offered, and I had to use a support worker to get me home.” (M, 70+, urban)* |
| Hospital Size | - Only a few of participants mentioned hospital size as any sort of consideration, though these participants were split along preference for large or small facility. - Larger hospital preference related to access to “*the services I needed*” and were seen as better *resourced* to respond to an *emergency* providing “*piece of mind*”. - Smaller hospital preference was because “*There wasn’t hundreds of patients and stuff like that, so [all the staff] were very attentive*”, “*more safety because there’s less people*” and easier to *navigate* and find things. | - *“I felt it’s a larger [hospital, this is better] – because I’d tried other ones and they just didn’t have enough [services].” (F, 30-39, urban)* - *“And the thing that I've come to understand also is the private hospitals tend to be smaller. Bit like private schools, they tend to be a bit smaller and the less people, the less sickness, the less issues, you know what I mean? And I think there's more safety because there's less people.” (F, 40-49, urban)* |
